# Prostate cancer stem cells: an updated mini-review

**DOI:** 10.7150/jca.100604

**Published:** 2024-10-28

**Authors:** Kaichen Zhou, Haosen Lu, Jielin Zhang, Qi Shen, Pengzhan Liu, Qiqing Xu, Chunhua Yang, Lijun Mao

**Affiliations:** 1 Department of Urology, the Affiliated Hospital of Xuzhou Medical University, Xuzhou 221000, China.; 2 Jiangsu Key Laboratory of Biological Cancer Therapy, Xuzhou Medical University, Xuzhou 221002, China.

**Keywords:** Stem cells, prostate cancer stem cells, surface markers, signaling pathway, therapy

## Abstract

Prostate cancer is the most common malignant tumor in male genitourinary system. The incidence of prostate cancer ranks the first among all male malignant tumors worldwide, and the mortality rate ranks the second among all male malignant tumors. Prostate stem cells are heterogeneous subsets with the function of self-regeneration and proliferation in the prostate, which can produce all cell lineages that make up the prostate epithelium. At present, the pathogenesis of prostate cancer remains unclear. According to cancer stem cell hypothesis, prostate cancer may be a stem cell disease, which provides a new direction for revealing the pathogenesis of prostate cancer and developing treatment strategy for prostate cancer. In this mini-review, we highlight recent advances in our understanding of the origin, surface molecular markers, signaling pathway and the significance for clinical treatment of prostate cancer stem cells.

## Introduction

Prostate cancer (PCa) is a highly heterogeneous tumor with multiple phenotypes of cancer cells. The heterogeneity, metastatic growth and drug resistance of cancer cells bring great challenges to the treatment of prostate cancer. Recent studies have shown that the key to the origin of cancer is to determine the cells of its origin, and cancer stem cells (CSC) may be the main cause of cancer to develop into invasive and metastatic diseases.

## Overview of prostate cancer stem cells

Stem cells are a group of cells with significant proliferation and self-renewal characteristics, and can differentiate into immature cells with specific cell potential in the tissue. Stem cells perform physiological functions such as maintaining tissue dynamic balance, tissue regeneration and repair, as well as proliferation and differentiation necessary for the normal operation of organs 2. Lapidot et al. 3 found for the first time that only a small number of leukemic tumor cells have the phenotype of CD34+CD38-, and these cells can form cancer in immunodeficient mice, and confirmed that these cells are tumor stem cells. Tumor stem cells (TSC), also known as cancer stem cells (CSC), are cells with the ability of self-renewal, unlimited proliferation and multi-differentiation potential, and they show invasive phenotypes and resistance for anticancer therapy 4,5. Prostate stem cells (PSC) can differentiate into at least three different types of prostate cells, namely basal cells, luminal epithelial cells and neuroendocrine cells. Similar to other types of cancer, PCa shows heterogeneity. Among different cell types, a small number of cell groups play a key role in the development of heterogeneous PCa and are defined as prostate cancer stem cells (PCSC). In this mini-review, we highlight recent advances in our understanding of the origin, surface molecular markers, signaling pathway and the significance for clinical treatment of PCSC. The readers can refer to the figures in recent reviews for better understanding of the concept of PCSC 6,7.

## The origin of PCSC

PCSC is similar to normal prostate stem cells, has a long life, remains in a state of resting or slow cycle, maintains low differentiation and has the characteristics of embryonic stem cells 6. There are three hypotheses about the source of PCSC: normal stem cells, transient expanded epithelial cells and terminally differentiated cavity cells. Current evidence supports the view that PCSC comes from normal prostate stem cells.

At present, the origin of prostate cancer is still controversial. Choi *et al.* 8 used pedigree tracking method to show that luminal epithelial cells are more likely to directly transform into malignant tumors after Pten knockout, while basal cells seem to differentiate into luminal epithelial cells with transforming function, and then transform into tumor cells. In the samples of prostatic intraepithelial neoplasia, the upregulation of proto-oncogene c-MYC and the shortening of telomere length were observed only in luminal cells, but not in basal cells 9. More than 95% of prostate cancer is acinar cancer. In this pathological type, basal cells rarely express androgen receptor (AR) and prostate specific antigen (PSA), while luminal cells express high levels of keratin 8 (K8), keratin (K18), AR and PSA 10-14.

However, some studies have confirmed that prostate basal cells are the origin cells of prostate cancer. Goldstein *et al.* 15 isolated basal cells and luminal epithelial cells from human benign prostatic hyperplasia, introduced tumor genes into both basal cells and luminal epithelial cells, and then transplanted them into mice. The results show that basal cells produce tumor tissue similar to human prostate cancer. In addition, overexpression of transcription factor ERG1 and fusion gene TMPRSS2 in mouse prostate basal cells/stem cells could lead to developmental disorders and prostatic intraepithelial neoplasia (PIN), but no similar phenotypes were found in luminal epithelial cells 16.

## Surface markers of PCSC

Reliable PCSC tags are essential for separating PCSC. Like many other CSC, PCSC is likely to have similar antigenic expression to its counterpart PSC 17. Accumulating evidence shows that PCSC expresses specific and non-specific surface markers (Table 1).

### Adhesion molecules CD24, CD44, CD133 and CD166

PCSC implanted into NOD/SCID mice could reconstruct tumor cell subsets and express CD44, CD133 and CD166 in different proportions. CD44 is a hyaluronic acid-bound cell surface glycoprotein that is expressed on endothelial cells and other cell surfaces. CD44 is a marker of early progenitor cells and participates in cell-to-cell adhesion, migration and interaction. Liu *et al.* 19 proved that CD44+ human prostate cancer cells can reconstruct tumors in immunodeficient mice by implanting human prostate cancer cells into the mice. It has been confirmed that CD44 is significantly overexpressed in stem cell spheres formed by prostate cancer cell lines 20. However, a splice variant subtype CD44v7-10 appears in cancer and is being studied as a more specific PCSC marker 21, 22. Because CD44 is not specific, CD44 is usually supplemented by a variety of other markers to sort PCSC. Hurt *et al.* 23 have shown that CD44+CD24- can recognize very early progenitor cells of prostate cancer, or PCSC itself, but further confirmation is needed. CD133 is a kind of glycoprotein with molecular weight of 120 kD and has become a cell surface marker of CSC in many cancers, including prostate cancer. CD133 is expressed in both normal prostate and prostate cancer, and its expression is related to tumorigenesis 24. CD133+ prostate cancer cells have stronger proliferation ability than CD133- cells, and the inhibition of CD133 can increase the sensitivity of prostate cancer cells to anticancer drugs 25, 26. It is considered that CD133+CD44+ and CD133+CD44- PCSCs have the highest ability of platelet aggregation, which may be attributed to the increased expression of prothrombin 27. In turn, platelet-derived SDF-1 α stimulates PCSC invasion. CD166 is involved in the maintenance of stem/ progenitor cell function 28. Similar to CD133, CD166+ prostate cancer cells have stronger ability of regeneration and proliferation and can produce primitive tumor heterogeneity in xenografts, while knockout of CD166 could inhibit tumor growth 29.

### α2β1 integrin

α2β1 integrin is considered to be the marker of normal prostate stem cell compartment 30. Collins *et al.* successfully isolated high-purity CD44+/ integrin α2β1 CD133 + cells (cells co-expressing these three molecules) from primary cultured prostate cancer cells, and confirmed that these cells have multi-directional differentiation, clone formation, self-renewal and strong proliferation potential. CD44+/α2β1 CD 133 + population isolated from PCa patients has strong tumorigenicity *in vitro* 31, 32. However, these cells have not been studied for their ability to replicate the whole tumor *in vivo*, and α2β1 CD133 + spectrum could be used as a marker of PCa stem cell niche.

### Trop-2

Recently, trop-2 has been considered as a new marker for the identification of prostate stem cells in mice 33. Depending on the low or high expression level of trop-2, Lin-Sca1+CD49fin showed low or high stem cell-like activity in normal mouse prostate cells *in vitro* and *in vivo*. However, the expression of trop-2 in human prostate has never been studied.

### Stem cell antigen 1 (Sca1)

Sca1 is a phosphatidylinositol anchored protein of 18KD, which belongs to the Ly26 antigen family. It is expressed in stem/progenitor cells of some tissues, such as hematopoietic cells, myocardial tissue, mammary gland, skin, muscle and testis. Sca1+ cells show the characteristics of stem cells in prostate cancer and have the ability of multi-directional differentiation and proliferation. Sca1+ cells are replication-static and androgen-independent. Xin *et al.* 31 demonstrated that when androgen castration occurs, Sca1+ cells increase, and Sca1+ mouse prostate cells express PIN after lentivirus-mediated AKT1 overexpression, and then progress to PCa.

### ABCG2

Patrawala *et al.* 34 suggested that the expression of ABCG2 mainly marks rapidly circulating tumor progenitor cells. ABCG2+ cells are progenitor cells with multi-directional differentiation potential, and ABCG2- cells contain primitive stem cell-like cancer cells. ABCG2 expression is also regulated by many PCSC-related signaling pathways, such as Hedgehog, Notch and PTEN/PI3K/AKT pathways 35. Guzel *et al.* 36 found that the expression of stem-related genes Sox2, Oct4, Nanog and ABCG2 increased in recurrent prostate cancer tissue samples, indicating the enrichment of PCSC. Therefore, ABCG2 may not be an ideal marker for tumor stem cell sorting, but it is an important marker to identify tumor drug resistance.

### Aldehyde dehydrogenase (ALDH)

ALDH has been proposed as a marker of PCSC, and ALDH+ prostate cancer cells have the characteristics of CSC 37. Primary culture of human prostate cancer cell lines and clinical prostate cancer samples contained cell subsets with high ALDH enzyme activity, and ALDH+ prostate cancer cells showed enhanced tumorigenicity, clone formation and metastasis *in vitro* 38. At present, it is not clear to what extent the various ALDH subtypes contribute to high ALDH activity observed in highly tumorigenic and metastatic prostate cancer cells.

### TMPRSS2 gene

Studies have shown that 55% of prostate tumors have gene fusion (TMPRSS2:ERG) between prostate-specific TMPRSS2 gene and ERG oncogene, and confirmed the existence and expression of TMPRSS2:ERG in PCSC 39.

## Signaling pathways that regulate PCSC

### PTEN/PI3K/Akt signaling pathway

Dubrovska *et al.* 40 found that PCSC rich in CD133+/CD44+ has the possibility of causing tumor. Under the condition of ball growth, in addition to the increased protein levels of PI3K subunits p110 α and p110 β, the phosphorylation of several signal molecules involved in PI3K signal increased, including Akt1, GSK β, FoxO1a and FoxO3a. Knockdown of phosphatase and tensin homologue (PTEN) led to increased ball formation, and after the knockout of PTEN, PCSC significantly increased in prostate cancer cell lines. Prostate tissue can form carcinoma *in situ* and further develop into metastatic cancer. Similarly, the inhibition of PI3K activity by dual PI3K/MTOR inhibitor NVP-BEZ235 leads to the reduction of CD133+/CD44+ stem cell-like population in the prostate and inhibits the development of PCSC. Therefore, PTEN/PI3K/Akt pathway is very important for the maintenance of PCSC. In addition, a recent study demonstrated that mitogen-activated protein kinase (MAPK) signaling regulated androgen receptor signaling based on conditional deletion of PTEN in murine prostate epithelium 41.

### Notch signaling pathway

Notch signaling pathway is a key evolutionarily conserved pathway, and maintains the undifferentiated state of cells through paracrine inhibition to play an important role in cell development and differentiation. In recent years, more evidence shows that the abnormal activation of Notch is related to tumor progression. It has been found that abnormal activation of Notch signaling pathway can induce cell proliferation, metastasis and epithelial-mesenchymal transformation in many different solid tumors.

Notch is a heterodimer type I transmembrane receptor protein encoded by 1/4 Notch gene (Notch1-4). When Notch receptor binds to its ligand, it is activated to produce intracellular binding domain (ICN). The expression of ICN1 in stem cells and CSCs is upregulated, and Notch interacts with other signaling pathways such as PI3K/AKT, NF-κB and integrin signaling 42. Previous studies have shown that Notch signal elements such as Notch2, Notch3, Notch4 and Jagge-1 are expressed in LNCaP, C4-2B, DU145, PC3 and other prostate cancer cell lines. The level of ICN1-activated protein in PCSCs is significantly increased, and PCSC is much more resistant to chemotherapy than non-prostate cancer stem cells, indicating that Notch pathway is involved in the occurrence and development of PCa 43.

### Sonic hedgehog signaling pathway

Hedgehog (Hh) and its receptors Patched (PTCH) and Smoothened (SMO) play an important role in stem cell self-renewal, regeneration and tumor maintenance. The use of cyclodopamine or anti-Shh antibody to interfere with Shh-GLI pathway can inhibit the proliferation of primary prostate cancer culture of GLI+/PSA+, suggesting that this autocrine regulation of Shh signaling may be the key to maintain the growth of PCa 44. Model mice with overexpression of Hedgehog developed prostatic intraepithelial neoplasia (PIN) within 3 months, leading to the invasion and metastasis of PCa. In addition, androgen receptor (AR) was heterogeneously expressed during tumor progression, and the existence of P63 receptor AR -, CK14+/AR- and CD44+/AR- progeny suggested the direct acquisition of AR- malignant tumor traits 45. PCa is initiated by activating p63 + basal/stem cells and Hedgehog signaling pathway, suggesting that p63 gamma SMO + cells may develop to PCSC as carcinogenic cells. To sum up, the overexpression of Hedgehog leads to the formation of metastatic PCSC.

### Wnt/β-catenin signaling pathway

Wnt/β-catenin pathway is involved in many important cellular functions, such as stem cell regeneration and organogenesis 46. Wnt activity regulates self-renewal of prostate cancer cells, and their stem cell characteristics are independent of AR activity. Therefore, inhibition of Wnt/β-catenin pathway may play a role in reducing self-renewal of prostate cancer cells with stem cell characteristics. In addition, Song *et al.* 47 confirmed that mIR-1301-3p targeted Wnt pathway inhibitors Gsk3β and SFRP1, and promoted the proliferation of prostate cancer stem cells by inhibiting sFRP1 and GSK3β and activating Wnt/β-catenin pathway.

## The significance of clinical treatment of PCSC

About 40% of patients with localized prostate cancer relapsed after treatment. Although current treatments eliminate most tumors, they cannot eliminate CSC or tumor initiating cells (TICs). Therefore, the key to the origin of cancer is to determine the cells of its origin, and PCSC may be the main reason for the development of PCa into invasive and metastatic diseases. Some patients with PCa eventually develop hormone-resistant prostate cancer after androgen removal therapy. The ability of self-regeneration and metastasis of PCSC and the lack or low expression of androgen receptor may be the causes of drug resistance and castration-resistant prostate cancer in androgen deprivation therapy-resistant cells 48-50.

Indeed, a previous study reported that G-protein-coupled estrogen receptor enhanced the stemness of triple-negative breast cancer cells and promoted malignant characteristics 51. Further studies are needed to investigate the role of androgen receptor or other hormone receptor in the stemness of PCa cells.

Eradication of PCSC has been considered as a promising target to improve the prognosis of patients with advanced PCa. However, PCSC has ancestral drug resistance, and some inherent characteristics make it difficult to eradicate through traditional anticancer therapy. First of all, PCSC overexpresses some membrane transporters and has more powerful detoxification functions 52. Second, PCSC is at rest, while most anticancer drugs target proliferative cells 53. Third, PCSC has the ability to repair DNA damage efficiently 54.

More evidence shows that the specific markers of PCSC can predict the prognosis of patients, which provides a basis for PCSC as a potential clinical biomarker and therapeutic target 55. Some scholars have proposed therapeutic strategies for high expression of ABCG2 and telomerase in prostate cancer stem cells. ABC membrane transporter ABCG2 is highly expressed and telomerase level is significantly increased in prostate cancer stem cells. The growth of prostate cancer stem cells can be inhibited by inhibiting ABCG2 and telomerase activity 56. High affinity peptides targeting CD44v6 isoforms identified by phage display can be used for the diagnosis and systemic transmission of therapeutic molecules in patients with PCa 57.

In addition, the fact that PCSC participates in multiple signaling pathways creates the possibilities for new therapeutic strategies. The pathway involved in targeting PCSC can remove PCSC that is resistant to conventional therapy and improve the sensitivity of experimental tumor models to different treatments 58, 59. Wnt/β-Catenin signal pathway is cross-talked with Notch and Shh signaling pathway, which is of significance for cancer treatment and intervention 60. Targeting Notch/PI3K signaling pathway may be beneficial to the treatment of PCa by eliminating PCSC. Crude furcidotoxin can induce autophagy and apoptosis of prostate cancer stem cells through PI3K/Akt/mTOR signal pathway, thus inhibiting the progression of PCa 61. Hedgehog signaling pathway plays an important role in the transformation of normal prostate basal cells/stem cells into PCSCs, and hedgehog pathway inhibitor can induce apoptosis in human prostate cancer cell line PC3, which is poorly differentiated and highly tumorigenic after being transplanted into nude mice 43. NK cells can give priority to targeting tumor stem cells in a variety of solid tumors except PCa. It is confirmed that NK cells can preferentially target prostate cancer stem cell-like cells through the TRAIL/DR5 pathway, which may be of significance for the treatment and prognosis of PCa 62.

**Figure 1:**
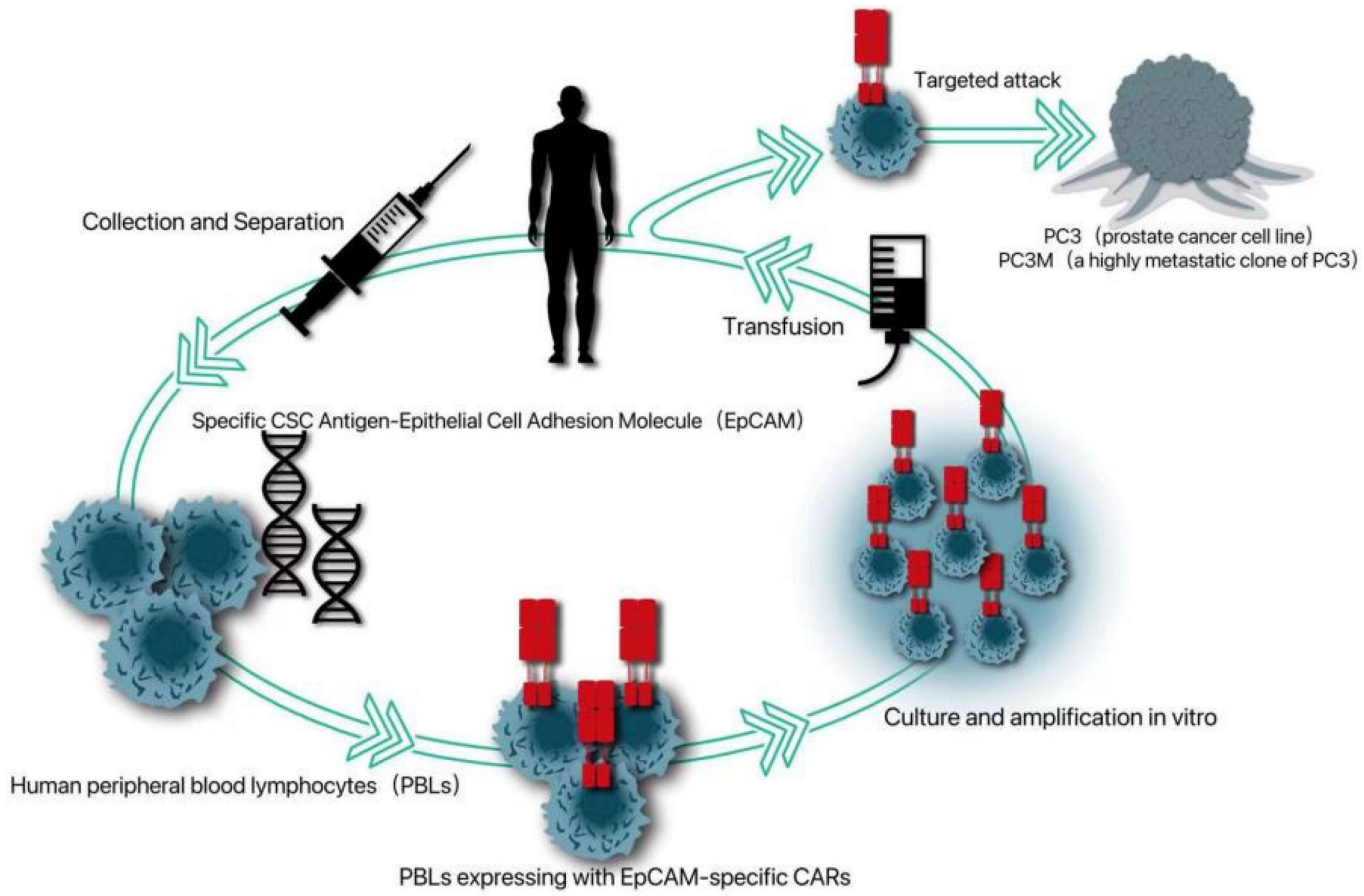
An EpCAM-specific chimeric antigen receptor (CAR) was constructed to transduce human peripheral blood lymphocytes (PBLs) and thereby enable them to target CSC marker EpCAM.

Because cancer patients can produce tumor-infiltrating lymphocytes with tumor-specific receptors, adoptive T cell therapy can be improved by introducing specific antigen receptors, namely tumor-specific T cell receptors or chimeric antigen receptors (CAR), into these circulating lymphocytes. Because CAR can induce T cells to attack tumors in an unrestricted way, CAR-T therapy has been applied to a variety of non-solid tumors, such as chronic lymphoblastic leukemia, lymphoma and neuroblastoma in hematological diseases 63. Because CSCs is more resistant to chemotherapy and radiotherapy, the possibility of immunotherapy for CSCs has been given more attention 64. Deng *et al.* introduced a specific CSC antigen-epithelial cell adhesion molecule (EPCAM)-specific CAR into human peripheral blood lymphocytes (PBL) 65. It was proved that PBL expressing CAR can not only kill PC3 tumors with high levels of EPCAM, but also significantly inhibit the growth of PC3 tumors with low EPCAM, which provides a new strategy for CAR-T therapy targeting PCSC (Fig. 1).

## Summary and prospects

PCa is still a big challenge for the society, especially with the aging of population 66. Fortunately, cancer stem cell theory has enabled us to have a better understanding of the mechanism of the occurrence and development of PCa, and help establish a new framework for clinical practice and basic research. In recent years, with the successful isolation and identification of prostate cancer stem cells, their specific and non-specific surface molecular markers and signal pathways have been discovered, which makes a further breakthrough in the diagnosis and treatment of PCa. However, due to the variability and complexity of PCa, there is still a long way to go before we can fully understand PCSC.

## Figures and Tables

**Table 1:** Surface markers of PCSC and their characteristics.

Marker	Other characteristics other than PCSC surface markers
CD24	CD24 is a glycosylphosphatidylinositol-anchored cell surface receptor expressed in a variety of cell types and tissues and can interact with a variety of ligands such as P-selectin and immune-related transmembrane protein Siglec family.
CD44	CD44 is a marker of early progenitor cells and a hyaluronic acid-bound cell surface glycoprotein. It is expressed on endothelial cells and other cell surfaces and participates in cell-to-cell adhesion, migration and interaction.
CD133	CD133 is a kind of glycoprotein with molecular weight of 120kD and five transmembrane regions, which has become a marker molecule for many kinds of tumor stem cells.
CD166	CD166 mediates cell adhesion and neutrophil migration.
α2β1 integrin	α 2 β 1integrin is essential for maintaining functional microenvironment.
Trop-2	Trop-2, a homologue of Trop-1/EpCAM, is expressed in epithelial-derived tissues and plays an important role in the early stages of tumor progression.
Sca1	Sca1 is a phosphatidylinositol anchored protein of 18KD, which belongs to the member of Ly26 antigen family and has the ability of multidirectional differentiation and proliferation.
ABCG2	ABCG2 is associated with multiple drug resistance of prostate cancer and is considered to be the molecular basis for sorting tumor stem cell-like cells resistant to chemotherapy.
ALDH	ALDH is a random tetramer responsible for catalyzing the oxidation of acetaldehyde to acetic acid.
TMPRSS2	TMPRSS2:ERG gene aberration may contribute to pT staging of prostate cancer.
